# Stage IIIC transitional cell carcinoma and serous carcinoma of the ovary have similar outcomes when treated with platinum-based chemotherapy

**DOI:** 10.4274/jtgga.2016.0190

**Published:** 2017-03-01

**Authors:** Gökhan Boyraz, Derman Başaran, Mehmet Coşkun Salman, Nejat Özgül, Kunter Yüce

**Affiliations:** 1 Department of Obstetrics and Gynecology, Division of Gynecologic Oncology, Hacettepe University Faculty of Medicine, Ankara, Turkey

**Keywords:** Epithelial ovarian cancer, serous papillary carcinoma, transitional cell carcinoma, platinum/taxane, Chemotherapy, cytoreduction

## Abstract

**Objective:**

Previous studies reported better outcomes for transitional cell carcinoma (TCC) of the ovary when compared with more common histologic types such as serous epithelial ovarian cancers (EOCs). The aim of this study was to compare the survival outcomes of platinum- based chemotherapy in patients with stage IIIC TCCs and serous EOCs.

**Material and Methods:**

Clinicopathologic features and survival data of patients with FIGO stage IIIC TCC and serous EOC who had undergone primary surgery followed by six cycles of intravenous platinum/taxane between 2007 and 2015 were retrieved from the database of Hacettepe University Hospital.

**Results:**

We identified 14 (10.9%) TCCs and 114 (89.1%) serous EOCs. The median follow-up duration was 28 months (range, 3-101 months). Univariate analysis revealed that the TCCs and serous EOCs had similar progression-free survival (PFS) and overall survival (OS). Patients with residual disease less than 1 cm had longer OS than patients with residual disease greater than 1 cm (75.0 vs. 45.0 months, p=0.012). Cox regression analysis of all potential prognostic factors showed that the only independent prognostic factor significantly associated with OS was residual disease less than 1 cm [hazard ratio=0.38; 95% confidence interval: (0.19-0.77); p=0.007].

**Conclusion:**

Surgically treated advanced stage TCCs did not have a significantly better prognosis after platinum/taxane-based chemotherapy when compared with serous EOCs. Residual tumor volume after primary surgery was the only independent predictor of OS in patients with EOC. Our results demonstrate the significance of achieving optimal cytoreduction in all histologic subtypes of EOC.

## INTRODUCTION

Ovarian transitional cell tumors include pure transitional cell carcinomas (TCCs) and Brenner tumors. TCCs are high-grade carcinomas of surface epithelial origin, as distinct from benign, malignant or borderline Brenner tumors. Primary TCC of the ovary is a rare subtype of epithelial ovarian cancer (EOC), which was first described by Austin and Norris in 1987 as a neoplasm composed of epithelial elements resembling urothelium and lack of a benign or borderline Brenner tumor (1, 2).

Transitional cell tumors, including pure TCCs and Brenner tumors of the ovary, represent approximately 2% of all ovarian tumors, and the pure form accounts for 1% of surface epithelial tumors (3, 4). The clinical presentation is not different from other types of ovarian carcinoma, and most common presenting symptoms are abdominal distension and pain (3). The primary treatment of patients with TCC of the ovary is also similar in patients with other EOC, which consists of optimal surgical resection, followed by adjuvant platinum-based chemotherapy (5).

Although many previous studies reported that patients with TCCs had better prognoses compared with patients with all other types of ovarian carcinomas following standardized surgery and chemotherapy (3, 5, 6), results are conflicting regarding the prognosis of TCCs. Therefore, the aim of this study was to compare the survival outcomes of patients with similarly treated stage IIIC transitional cell and serous EOCs.

## MATERIAL AND METHODS

Clinicopathologic and outcome data of patients with International Federation of Gynecology and Obstetrics (FIGO) stage IIIC TCCs and serous EOC who had undergone primary surgery followed by six cycles of intravenous platinum/taxane between 2007 and 2015 were retrieved from the database of Hacettepe University Hospital. All operations were performed by gynecologic oncologists. All patients underwent total abdominal hysterectomy, bilateral salpingo-oophorectomy, and pelvic and para-aortic lymphadenectomy with omentectomy and other cytoreductive procedures to achieve optimal cytoreduction. The clinical and pathologic characteristics of patients with TCCs and serous EOC including age, histologic subtype, stage, grade, preoperative CA-125 level, and survival were determined and compared.

Overall survival (OS) was calculated from time of diagnosis until death or last follow-up. Progression-free survival (PFS) was calculated from time of diagnosis until disease recurrence. The Kaplan-Meier survival analysis was used to estimate OS and PFS, and survival differences were analyzed using the log-rank test. Cox regression analysis was performed to assess the potential inﬂuence of other prognostic factors. Mann-Whitney U test, Chi-square test or Fisher's exact test were used as appropriate. Statistical analyses were performed using Statistical Package for the Social Sciences statistical software (version 16.0, SPSS Inc, Chicago, IL, USA). Differences were considered statistically significant at p<0.05. Local Ethics Committee permission was not sought because this study represents a retrospective database review.

## RESULTS

We identified 14 patients with stage IIIC TCCs who were treated between 2004 and 2015 at Hacettepe University Hospital. These 14 patients’ results were compared with 114 patients with stage IIIC serous EOC. The median ages of patients with TCC and serous EOC was 55 and 57 years, respectively. The median age, CA-125 level at diagnosis, and grade were similar for both groups. Optimal resection to <1 cm residual disease was achieved in 50% of patients with TCCs and 60.5% of patients with serous EOCs and there was no difference in the rates of optimal cytoreduction between the two groups. Histologically, all of the TCCs were grade 3. However, of the serous EOCs, 20 (17.5%) were grade 1-2 and the remainder (82.5%) was grade 3. All patients received 6 cycles of paclitaxel and carboplatin chemotherapy. Demographic, clinical, and pathologic characteristics of the study patients are presented in Table 1.

**Figure 1:**
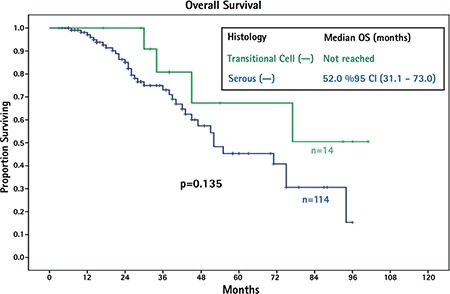
Median overall survival curves based on tumor histology

**Figure 2:**
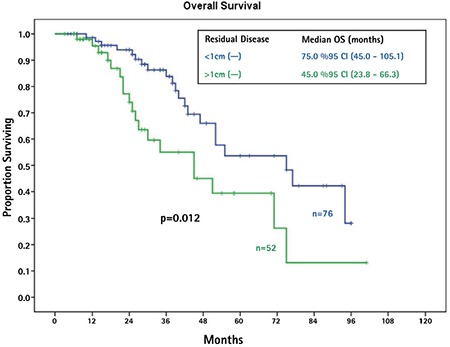
Median overall survival curves based on residual tumor volume after initial surgery in the whole study cohort

The median follow-up duration was 28 months (range, 3-101 months). The median OS of patients with TCCs was not obtained because <50% of patients died of disease at the time of analysis. Patients with serous EOCs had a median OS of 52 months. The median OS was not significantly different between the two groups (p=0.135). The median PFS for TCCs was 15 months, and patients with serous EOCs had a median PFS of 21 months (p=0.242). In substance, univariate analysis revealed that TCCs and serous EOCs had similar PFS and OS (Figure 1). Patients with residual disease less than 1 cm had longer OS than patients with residual disease greater than 1 cm (75.0 vs. 45.0 months, p=0.012). Figure 2 presents the median OS curves based on residual tumor volume in the whole study cohort. Cox regression analysis of potential prognostic factors showed that the only independent prognostic factor significantly associated with OS was residual disease less than 1 cm [hazard ratio=0.38; 95% confidence interval: (0.19-0.77); p=0.007] (Table 2).

## DISCUSSION

Limited data suggests that TCC is more chemosensitive and is associated with a better prognosis than serous carcinoma of the ovary (7, 8, 9). Austin and Norris (2) who first described TCC of the ovary, also reported a better response to chemotherapy in patients with TCC. Kommoss et al. (6) found that TCCs had a significantly better prognosis when compared with all other types of ovarian carcinomas after standardized chemotherapy. However, they also documented that this better prognosis could be related with better surgical resectability and less tendency to the large extraovarian tumor spread of TCCs. In addition, they performed a subgroup analysis in patients with postoperative residual disease <1 cm; there was no statistical significant survival difference among patients with TCCs and other types of ovarian carcinomas (6). Guseh et al. (10) reported that TCCs of the ovary had a significantly lower risk of platinum resistance and also had improved overall survival when compared with patients with serous EOCs. In contrast to Kommoss et al. (6) study, they found a similar rate of surgical resectability between TCCs and controls (10). Robey et al. (9) performed a retrospective study and reported that although patients with non-TCC predominant tumors had a higher percentage of tumor recurrences after chemotherapy, most TCC predominant tumors responded completely to chemotherapy even if residual tumor was ≥2 cm. Similarly, Silva et al. (8) reported that TCCs of the ovary had an excellent response to different chemotherapy regimens.

It has been suggested by other authors that TCCs of the ovary have no significant difference in survival outcomes compared with serous EOCs after adjuvant chemotherapy. Hollingsworth et al. (11) evaluated 58 patients with EOC. Of these patients, 13 had TCC, 2 had mixed histology, 25 had serous ovarian cancer, and the remaining 18 patients had other types of EOC. The authors found that TCC histologic subtype was not a significant predictor for OS or DFS and concluded that TCC did not provide a favorable prognosis (11). Mackay et al. (12) performed a meta-analysis to evaluate the prognosis of women with rare EOC histology. Thirty-six patients with TCC were included in their study. When controlled for prognostic factors as age, stage, and residual disease, patients with TCC did not significantly differ from those with serous carcinoma. In addition, they found that patients with transitional cell histology were more likely to be optimally debulked to no visible tumor compared with those with serous histology after surgery (12). Consistent with this study, our data suggests that TCCs have a similar OS or PFS after platinum/taxane-based chemotherapy when compared with serous EOCs. However, we found a similar rate of surgical resectability between TCCs and controls [50% vs. 60.5% (p>0.05), respectively]. Additionally, consistent with previous studies, residual tumor volume after primary surgery was the only independent predictor of OS in patients with EOC in the present study.

There are different results in the literature related with the prognosis and response to chemotherapy in patients with TCC of the ovary. These differences can be associated with lack of prospective studies due to the small number of patients. Other possible related factors include differences in pathologic diagnostic criteria and surgical practice patterns of individual oncology centers. Furthermore, some of the studies were published before the introduction of taxanes, which are routinely used in primary adjuvant therapy in patients with EOC. On the other hand, recent molecular and immunohistochemical data demonstrated that TCCs express the same immunophenotype and genetic mutations as high-grade serous carcinoma (13, 14). For instance, Cuatrecasas et al. (1) found that TCCs of the ovary showed p16 and p53 overexpression, and p53 mutations as in other high-grade ovarian carcinomas (1). These findings support that TCC could be a variant of serous EOC. Our data suggest that TCCs have a similar clinical and prognostic behavior as serous EOC. However, further research is needed to demonstrate the clinical and pathologic similarities between TCCs and serous EOCs. From a practical point of view, the results of our study strongly support the most important surgical oncology doctrine, which is to achieve no visible disease in patients with peritoneal carcinomatosis of epithelial ovarian origin. This principle should guide gynecologic oncologists until further robust data becomes available in the era of targeted therapy, which may nullify the heroic actions of dedicated surgeons.

Our study has inherent limitations due to its retrospective study design. The presence of other possible confounding variables such as selection and recall bias, which might have affected our results, cannot be ruled out because of the retrospective nature of the study. Another disadvantage of the present study is its limited sample size. Therefore, any conclusions maybe limited in their implications. Despite these limitations, our study adds to significant findings because of our specific group of stage IIIC TCCs.

In conclusion, TCC of the ovary is a rare type of EOC. Surgically treated advanced stage TCCs did not have a significantly better prognosis after platinum/taxane-based chemotherapy when compared with serous EOCs. Residual tumor volume after primary surgery was the only independent predictor of OS in patients with EOC. Our results demonstrate the significance of achieving optimal cytoreduction in all histologic subtypes of EOC. Furthermore, our study suggests that TCC does not relate with a favorable prognosis or better response rate to chemotherapy.

## Figures and Tables

**Table 1:**
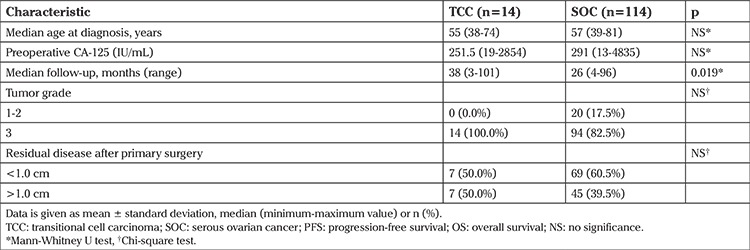
Patient characteristics in the study cohort

**Table 2:**
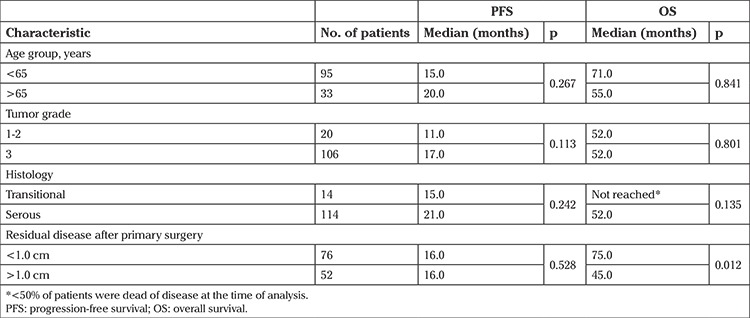
Median progression-free survival and overall survival based on potential prognostic factors
